# In Vitro and In Vivo Cardioprotective Effects of Curcumin against Doxorubicin-Induced Cardiotoxicity: A Systematic Review

**DOI:** 10.1155/2022/7277562

**Published:** 2022-02-21

**Authors:** Qian Zhang, Lei Wu

**Affiliations:** ^1^Department of Pharmacy, The Bozhou Hospital, Anhui Medical University, Bozhou Anhui 236800, Bozhou 236800, Anhui, China; ^2^Pharmacy Department, Hua Tuo Traditional Chinese Medicine Hospital, Bozhou 236800, Anhui, China

## Abstract

**Objective:**

This study aimed to review the potential chemoprotective effects of curcumin against the doxorubicin-induced cardiotoxicity.

**Methods:**

According to the PRISMA guideline, a comprehensive systematic search was performed in different electronic databases (Web of Science, PubMed, and Scopus) up to July 2021. One hundred and sixty-four studies were screened in accordance with a predefined set of inclusion and exclusion criteria. Eighteen eligible articles were finally included in the current systematic review.

**Results:**

According to the in vitro and in vivo findings, it was found that doxorubicin administration leads to decreased cell survival, increased mortality, decreased bodyweight, heart weight, and heart to the bodyweight ratio compared to the control groups. However, curcumin cotreatment demonstrated an opposite pattern in comparison with the doxorubicin-treated groups alone. Other findings showed that doxorubicin significantly induces biochemical changes in the cardiac cells/tissue. Furthermore, the histological changes on the cardiac tissue were observed following doxorubicin treatment. Nevertheless, for most of the cases, these biochemical and histological changes mediated by doxorubicin were reversed near to control groups following curcumin coadministration.

**Conclusion:**

It can be mentioned that coadministration of curcumin alleviates the doxorubicin-induced cardiotoxicity. Curcumin exerts these cardioprotective effects through different mechanisms of antioxidant, antiapoptosis, and anti-inflammatory. Since the finding presented in this systematic review are based on in vitro and in vivo studies, suggesting the use of curcumin in cancer patients as a cardioprotector agent against cardiotoxicity mediated by doxorubicin requires further clinical studies.

## 1. Introduction

For many years, cardiovascular anomalies are considered as the leading cause of death in the worldwide; therefore, cardiotoxicity has become a crucial concern for scientific community [1, 2]. Literature supports that severe cardiotoxicity is commonly evident in antitumor treatments [3–8]; hence, even if these patients survive from malignant tumor, they end up with heart complications in longer run that lead to compromised life style or death [9]. In this regard, drug-induced cardiotoxicity has withdrawn much attention in past two decades, as it has led to ban on various classes of drugs (such as rosiglitazone, prenylamine, rofecoxib, and levomethadyl acetate) due to their associated severe cardiotoxicity [10–13]. Nevertheless, there are still some cardiotoxic drugs that are applied by clinicians either other safer alternatives are not available or these drugs outweigh the risk of cardiac deformities.

Doxorubicin (adriamycin) is an effective chemotherapy drug which belongs to nonselective class I anthracycline family [14]. This chemotherapeutic agent is widely applied for the treatment of different cancers, such as acute leukemia, lymphomas, lung cancer, testicular cancer, thyroid cancer, ovarian cancer, breast cancer, and so on [15–19]. Major limitation reported for doxorubicin is the associated toxicity on various body organs, particularly the heart [9]. The doxorubicin-induced cardiotoxicity can manifest as aberrant arrhythmias, congestive heart failure, and ventricular dysfunction [20–22]. It has been also reported that the doxorubicin-induced cardiomyopathy has poor prognosis and can lead to death in most of the cases [17, 23]. Fortunately, the mentioned adverse effects overcome through combination chemotherapy [24, 25]. In this regard, coadministration of doxorubicin with other agents having chemoprotective capabilities can diminish the toxicity to normal tissues and enhance the tumoricidal efficacy of doxorubicin at the same time [26, 27].

According to the published studies, it can be stated that using the herbal and natural compounds or their derivatives to alleviate the adverse effects induced by radiotherapy/chemotherapy agents (radio/chemoprotectors) and/or increase the sensitivity of tumoral cells to radiotherapy/chemotherapy agents (radio/chemosensitizers) has attracted much attention over the past several decades [28–31]. Curcumin is a vibrant yellow spice extracted from rhizome of turmeric (*Curcuma longa*) that is insoluble in water [32, 33]. This natural polyphenol is a main active component of turmeric [34] that has antioxidant [35, 36], antiapoptotic [37], anti‐inflammatory [38–40], hepatoprotective [41, 42], analgesic and antiarthritic [43, 44], pulmonoprotective [45], lipid‐modifying [46, 47], immunomodulatory [48, 49], and antidiabetic [50, 51] actions. Curcumin also has anticancer activity, and it has been assessed in different malignant tumors, including colorectal cancer, prostate cancer, lung cancer, gastric cancer, breast cancer, and so on [52]. Moreover, this herbal agent can be used as an adjuvant in combination with other cancer therapeutic modalities such as radiotherapy and chemotherapy [32, 53]. In this regard, curcumin is able to alleviate the radiotherapy/chemotherapy-induced adverse effects (radio/chemoprotectors) and/or increase the sensitivity of cancer cells to radiotherapy/chemotherapy drugs (radio/chemosensitizers) which exerts the mentioned effects through the antiproliferative, antioxidant, antiapoptotic, and anti-inflammatory activities.

To the best of our knowledge, the present study is the first systematic review on the cardioprotective effects of curcumin, as an adjuvant, against doxorubicin-induced cardiotoxicity. In this regard, we tried to answer the following questions: (a) How does doxorubicin chemotherapy drug lead to cardiac adverse effects? (b) What are the underlying mechanisms of cardiotoxicity induced by doxorubicin? (c) What is the role of curcumin on the doxorubicin-induced cardiotoxicity? (d) What are the cardioprotective mechanisms of curcumin against doxorubicin-induced cardiotoxicity?

## 2. Methods

In this study, a systematic search was done in accordance with the Preferred Reporting Items for Systematic Reviews and Meta-Analyses (PRISMA) guideline [54]. Furthermore, a PICO framework was used to structure the review process [54]. This framework includes participants (P): cardiac cells damaged by doxorubicin (in vitro studies) and/or patients/animals with cardiac adverse effects induced by doxorubicin (clinical/in vivo studies); intervention (I): patients/animals/cells treatment with doxorubicin chemotherapeutic drug; comparison (C): patients/animals/cells treated with curcumin and doxorubicin; outcomes (O): there were two critical outcomes: changes induced in the cardiac cells/tissue following doxorubicin treatment compared to control/untreated groups and changes resulted in the cardiac cells/tissue following combined treatment of curcumin and doxorubicin in comparison with doxorubicin treatment alone.

### 2.1. Search Strategy

We performed a comprehensive systematic search for obtaining all relevant studies on “the role of curcumin on doxorubicin-induced cardiotoxicity” in both medical subject heading (MeSH) or advance in the electronic databases of Scopus, PubMed, and Web of Science up to July 2021 using the keywords of “Doxorubicin” OR “Adriamycin” AND “Curcumin” AND “Heart” OR “Cardiac” OR “Cardiac Toxicity” OR “Cardiac Toxicities” OR “Cardiomyopathy” OR “Arrhythmias” OR “Myocardium” OR “Myocardial” OR “Myocyte” OR “Cardiomyocyte” OR “Cardiopathic” OR “Cardiopathy” OR “Cardiotoxicity” OR “Cardiotoxicities” in title, abstract, or keywords.

### 2.2. Process of Study Selection

The inclusion criteria considered in this systematic review were full-text scientific studies with English language; our per-defined aim on the role of curcumin on doxorubicin-induced cardiotoxicity (based on the aforementioned keywords); adequate findings; no restriction in publications with clinical, in vivo, or in vitro studies; and no restriction on publication year. Additionally, the exclusion criteria considered for this study were hemodynamic data, not related articles, review papers, case reports, book chapters, letters to the editors, oral presentations, posters, and editorials.

### 2.3. Process of Data Extraction

Each eligible study was evaluated by two researchers, and the following data were then extracted: author name and year of publication; models (clinical, in vivo or/and in vitro); doxorubicin dosage, protocol of usage, and administration route type; outcomes of doxorubicin on cardiac cells/tissue; curcumin dosage, protocol of usage, and administration route type; and curcumin coadministration outcomes.

## 3. Results

### 3.1. Literature Search and Screening

**Figure 1:**
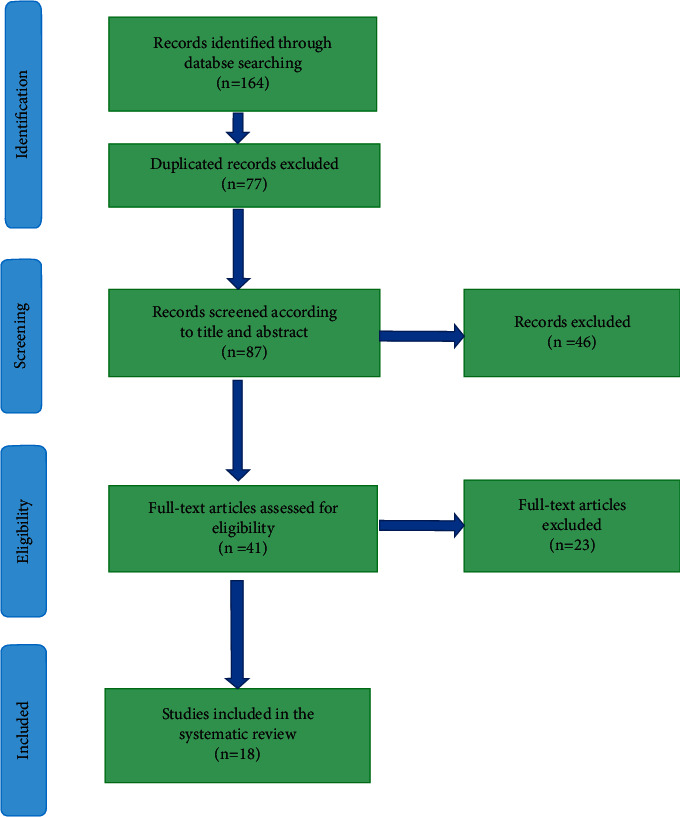
Flow diagram of PRISMA applied in the current study for the selection process.

The study selection process is shown in Figure 1.

One hundred and sixty-four articles were obtained by a comprehensive and systematic search on the electronic databases mentioned above up to July 2021. After removing the duplicated articles (*n* = 77), the remaining ones (*n* = 87) were screened in their titles and abstracts. After that, 46 articles were excluded, and remaining ones (*n* = 41) were qualified for evaluation of their full-texts. Eighteen articles were finally included in this systematic review based on the abovementioned inclusion and exclusion criteria.  Table 1 provides a summary of the findings extracted from the eligible articles.

### 3.2. The Cardioprotective Effects of Curcumin against Doxorubicin-Induced Cardiotoxicity

#### 3.2.1. Cell Survival and Mortality

According to the in vitro results, it was found that the cardiac cell survival following doxorubicin treatment was significantly lower than the untreated/control group [2, 9, 55–57]. Furthermore, the doxorubicin-mediated cytotoxicity was dose and time-dependent. It showed a direct relation between the cell death and posttreatment time/chemotherapy dosage [55, 56]. In contrast, the findings revealed that cotreatment of curcumin resulted to protect the cardiac cells against doxorubicin-induced decrease in cell viability [2, 9, 57]. However, a number of studies have shown that combined treatment of curcumin and doxorubicin leads to a further reduction in cell viability than doxorubicin-treated groups alone [55, 56].

The results of in vivo studies demonstrated that the mortality in doxorubicin-treated rats was significantly more than the untreated/control group [58–60]. The use of curcumin significantly decreased doxorubicin-induced mortality [58–60]. For instance, Imbaby et al. reported the survival rates of 58.33%, 66.33%, and 91.66% in the animals treated by doxorubicin, doxorubicin plus curcumin 100 mg/kg, and doxorubicin plus curcumin 200 mg/kg [59].

#### 3.2.2. Changes in Bodyweight and Heart Weight

Some studies showed that the bodyweight and heart weight and ventricle weight, and volume of animals treated by doxorubicin were less than the control group [58–63]. It was also reported that ratio of heart to the bodyweight of mice/rats were reduced following doxorubicin administration than the control group [60, 63].

Coadministration of curcumin and doxorubicin to the mice/rats led to increase the bodyweight, heart weight, ventricle weight and volume, and ratio of heart to bodyweight in comparison with the doxorubicin-treated groups alone [58–63].

#### 3.2.3. Biochemical Changes

The biochemical changes induced on heart cells/tissue can be observed following the doxorubicin treatment, as given in Table 1. For instance, some studies have been reported that the reactive oxygen species (ROS), lactate dehydrogenase (LDH), aspartate aminotransferase (AST), creatine kinase, lipid peroxide (LPO), serum glutamate oxaloacetate transaminase (SGOT), 8-OHdG, malondialdehyde (MDA), thiobarbituric acid reactive substances (TBARS), alanine aminotransferase (ALT), alkaline phosphatase (ALP), nitric oxide (NO), inducible nitric oxide synthase (iNOS), Bcl-2-associated X protein (BAX), BAX to B cell lymphoma 2 (Bcl-2) ratio, caspases-1, 2, 3, and 9, creatine phosphokinase (CPK), creatine kinase-myocardial band (CK-MB), nuclear factor-kappa B (NF-*κ*B), cyclooxygenase-2 (COX-2), LC3II to LC3I ratio, Beclin1, troponin I, troponin-T, interleukin 1 beta (IL-1*β*), IL-6, IL-18, tumor necrosis factor alpha (TNF-*α*), interferon gamma (INF-*γ*), monocyte chemoattractant protein-1 (MCP-1), early growth response protein-1 (Egr1), and NLR family pyrin domain containing 3 (NLRP3) levels were significantly increased in the doxorubicin-treated groups compared to the control groups [2, 55–70]. In contrast, the glutathione peroxidase (GPx), glutathione (GSH), mammalian target of rapamycin (mTOR) phosphorylation, and phospho-Akt levels were significantly reduced following the doxorubicin administration compared to the control groups [57–60, 63, 65, 66, 68–70].

The findings also showed that the curcumin cotreatment alleviated doxorubicin-induced biochemical alterations on heart cells/tissue (for most of the cases) [2, 56–70]. Nevertheless, it was found that the curcumin cotreatment synergized the effects of doxorubicin on several biochemical markers (for instance, ROS, superoxide dismutase (SOD), BAX, Bcl-2, and caspases-2, 3, and 9) [2, 55–57].

As given in Table 1, there were conflicting findings on several biochemical markers. In this regard, several studies reported the elevated levels of catalase, SOD, and Bcl-2 following doxorubicin treatment alone [2, 57, 65], while other studies reported the decreased levels for these biomarkers [2, 56–60, 62, 66, 68, 70]; however, combined treatment of curcumin and doxorubicin alleviated the changes induced by doxorubicin on these biochemical markers [2, 57–60, 62, 65, 66, 68, 70].

#### 3.2.4. Histological Changes

The obtained data from histological assessment of the doxorubicin-treated mice/rats showed the following histological changes: loss of myofibrils, cytoplasmic vacuolization, degeneration of cardiac muscle fibers, interstitial edema, increase in percentage of injured cells, reduction in cardiomyocyte population, severe hemorrhage, inflammatory cell infiltration, cellular swelling, spotted necrosis, interstitial cell hypertrophy, atrophic changes in myocardium and vessels, fibrous tissue formation, wavy degeneration of cardiac muscle fibers, damaged mitochondria, and so on [2, 57–62, 68].

The results of most studies demonstrated that coadministration of curcumin and doxorubicin could alleviate the doxorubicin-induced histological changes [2, 57–62, 68].

## 4. Discussion

**Figure 2:**
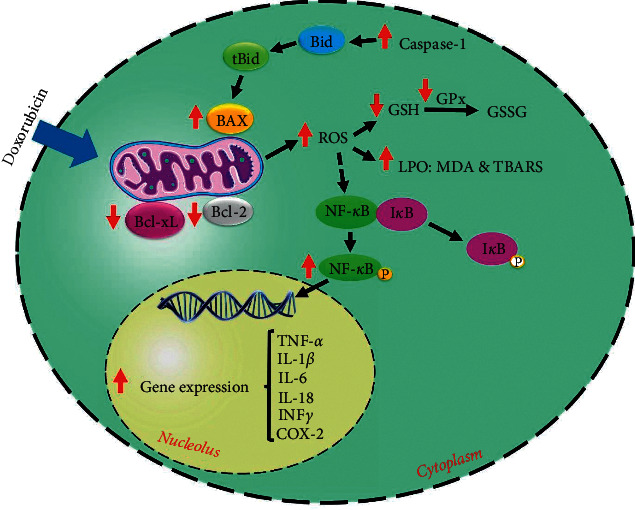
The molecular mechanisms of cardiac adverse effects mediated by doxorubicin. Mostly, doxorubicin induces oxidative stress through mitochondrial dysfunction. This chemotherapy agent increases free radicals via inhibition of GSH and GPx and also elevates LPO markers (MDA and TBARS). Furthermore, doxorubicin increases the apoptosis process through increments in BAX and caspase-1 activities. Moreover, doxorubicin elevates the inflammatory mediators (such as NF-*κ*B, COX-2, TNF-*α*, INF-*γ*, IL-1*β*, IL-6, and IL-18) leading to the cell injury. Curcumin, through an opposite pattern (antioxidant, antiapoptotic, and anti-inflammatory activities), alleviates these doxorubicin-induced cardiac adverse effects. ↑, increased by doxorubicin; ↓, decreased by doxorubicin; BAX, Bcl-2-associated X protein; GSH, glutathione; GPx, glutathione peroxidase; IL-1*β*, interleukin 1 beta; LPO, lipid peroxidation; MDA, malondialdehyde; TBARS, thiobarbituric acid reactive substances; NF-*κ*B, nuclear factor-kappa B; COX-2, cyclooxygenase-2; INF-*γ*, interferon gamma; TGF-*β*1, transforming growth factor beta 1; and TNF-*α*, tumor necrosis factor alpha.

In the current study, we aimed to review the doxorubicin-induced adverse effects on the cardiac cells/tissue. Additionally, the coadministration effects of curcumin on these adverse effects were reviewed. The findings obtained from the effects of doxorubicin treatment alone or in combination with curcumin on the cardiac cells/tissue are given in Table 1. Moreover, some of the important changes on the cardiac cell following doxorubicin administration as well as the effects of curcumin coadministration on these changes are shown in Figure 2.

Doxorubicin, a chemotherapeutic drug belonging to the anthracycline group, is highly effective in the treatment of a variety of malignant tumors [71]. The mechanisms of action for doxorubicin cytotoxic effects in cancerous cells include DNA intercalation leading to topoisomerase II disruption as well as generation of ROS resulting in cell membrane and mitochondrial membrane damage [17, 72]. Nevertheless, the clinical use of doxorubicin is restricted by the risk of dose-dependent cardiotoxicity [62]. A variety of mechanisms for explanation of doxorubicin-induced cardiomyopathy have been proposed, including oxidative stress, inflammation, apoptosis, iron-loading disorders, and calcium dysregulation [73]. It has been suggested that the use of chemoprotective agents (such as curcumin) can mitigate the doxorubicin-induced cardiotoxicity.

The antitumoral activity of curcumin has been reported in some cancers [52]. Moreover, this natural phytochemical agent has multiple biological activities including chemosensitizing properties [74, 75] and chemoprotection effects [76, 77]. Curcumin exerts its chemoprotective effects through its antioxidant, antiapoptotic, anti-inflammatory activities, and so on. In the following subsections, the mechanistic effects of the doxorubicin chemotherapeutic agent on the cardiac cellular pathway and the mechanistic effects of curcumin against the cardiac adverse effects induced by doxorubicin are discussed.

### 4.1. Antioxidant Actions

Normally, free radicals are produced in the cells in which the relevant defense mechanisms protect the cells against them [78, 79]. Of note, the free radicals increase in oxidative stress conditions because an imbalance between the free radicals and these defense systems occurs [80, 81]. Some studies have reported that the doxorubicin administration increases the ROS level of cardiac cells. The ROS attacks the cell macromolecules and leads to malfunction of the heart tissue [2, 56, 57, 64]. It also showed that upon mitochondrial damage, the generation of free radicals increases in the cells [82]; in this regard, doxorubicin through impairment of mitochondrial function can elevate the generation of free radicals [2, 64, 83]. Moreover, this chemotherapeutic agent is able to increase the LPO level and decrease the GPx and GSH levels in the heart cells which lead to the cell membrane devastation and malfunction [57–60, 65, 66, 68–70]. Hydrogen peroxide (H_2_O_2_), as a nonradical ROS, produces 2H_2_O via the activity of GPx enzyme and consuming GSH [84]. According to these findings, it can be mentioned that doxorubicin impairs free radical scavenging capacity of intracellular antioxidant enzymes. Furthermore, there is normally a low amount of NO in the cardiac cells [85] in which its level is increased following doxorubicin treatment [59]. It is noteworthy that NO has remarkable roles in cellular signaling during pathological processes [86, 87]. It was also found that doxorubicin leads to increase the O_2_^−^ level in cardiac cells [63]. Interaction of NO with O_2_^−^ generates ONOO^−^ that is a potential free radical [88]. The ONOO^−^ can also turn to NO_2_^−^, NO_3_^−^, OH^−^, and CO_3_^−^. Therefore, O_2_^−^ can induce generation of reactive nitrogen species (RNS) [85]. Another study has reported increased expression of 8-OHdG and 3,3′-dityrosine (as biomarkers of oxidative damage) in the hearts of the rats treated by doxorubicin [68].

It has been shown that curcumin through its antioxidant effects can help scavenging of free radicals generated by some chemotherapeutic agents, resulting to ameliorate toxic effects of chemotherapy [89]. The results represented in the current study revealed that curcumin could decline the doxorubicin-elevated ROS level of cardiac cells [57, 64]. However, it was also found that this herbal agent had a synergistic effect on the doxorubicin-generated ROS level in a concentration-dependent manner [56]. Moreover, Jain and Rani [2] reported that curcumin response on the doxorubicin-induced ROS level in cardiomyoblasts was dependent on mode of treatment, as the cells pretreated with curcumin for one day followed by doxorubicin treatment could decrease the doxorubicin-generated ROS level, while it was increased in the cells simultaneously treated with curcumin and doxorubicin [2]. This antioxidant agent could also reduce doxorubicin-induced mitochondria injury, thereby, inhibiting doxorubicin-induced ROS generation [64]. Moreover, the increased levels of NO and O_2_^−^ following doxorubicin treatment were declined by the curcumin cotreatment [59, 63]. Furthermore, the combined treatment of curcumin and doxorubicin (compared to doxorubicin treatment alone) could upregulate GSH and GPx expressions [57–60, 65, 66, 68–70] and downregulate MDA, TBARS, 8-OHdG, and 3,3′-dityrosine levels in the cardiac cells [57, 58, 60, 63, 65, 68, 70].

### 4.2. Antiapoptotic Actions

Apoptosis (or programmed cell death) induced by doxorubicin is beneficial in cancer treatment; nevertheless, the apoptotic effect of this chemotherapeutic agent in cardiac cells is responsible for its cardiotoxicity [90]. Some important mediators involved in the apoptosis process are caspase enzymes, cleaved poly (ADP-ribose) polymerase (PARP), Bcl-2, p53, BAX, B cell lymphoma-extra large (Bcl-xL), NFAT5, and ceramide [85, 91–100]. Moreover, the apoptosis process can occur following the massive DNA damage and the oxidative stress conditions [101, 102]. Some studies have showed that the using doxorubicin elevates the apoptosis level in the cardiac cells compared to the control groups [56, 57, 62, 64, 70]. Doxorubicin can also downregulate Bcl-xL expression [103] and upregulate BAX, cleaved caspases-1, 2, 3, and 9, and p53 expressions in the cardiac cells than the untreated groups [2, 55–57, 62, 63, 68, 104, 105]. These findings demonstrate that the cells are moving towards apoptosis. Furthermore, doxorubicin impedes matrix metalloproteinase (MMP), mediates mitochondrial swelling, and opens mitochondrial permeability transition pore (mPTP), leading to apoptosis [106]. It has been also reported that doxorubicin can elevate PARP activity [107–109]. This nuclear enzyme (PARP) is able to regulate many cellular processes, including apoptosis, DNA repair, genomic stability, and chromatin functions [85, 110]. The findings from other studies showed that doxorubicin can trigger cardiac apoptosis via activation of p53, c-Jun N-terminal kinases (JNKs), and p38 mitogen-activated protein kinases (MAPKs) pathways [111].

Although curcumin can induce apoptosis in cancerous cells [112–115], it is also able to protect normal cells/tissues during cancer chemotherapy through its antiapoptotic effects [76, 116]. The data represented in this systematic review showed that combined treatment of curcumin and doxorubicin results to decrease the apoptosis level of cardiac cells in comparison with the doxorubicin-treated groups alone [57, 62, 64, 70]. It was also found that curcumin had a synergistic effect on the doxorubicin-induced apoptosis level in a dose-dependent manner [56]. Additionally, mode of treatment (as pretreatment or concomitant) affected the curcumin response to doxorubicin-induced apoptosis in cardiomyoblasts; in this regard, it was found that the cardiac cells pretreated with curcumin for one day followed by doxorubicin treatment showed a decrease in the apoptosis level induced by doxorubicin, while the apoptosis level was increased in the cells simultaneously treated with curcumin and doxorubicin [2]. The pretreatment of cardiac cells with curcumin followed by doxorubicin prevented loss of MMP and decreased mPTP opening [57]. Furthermore, curcumin combined to doxorubicin could modulate the expression of antiapoptotic (Bcl-2) and proapoptotic (BAX and caspases-1, 2, 3, 8, and 9) mediators in the cardiac cells treated by doxorubicin [2, 55–57, 62, 63, 68].

### 4.3. Anti-Inflammatory Actions

The chemotherapy treatment can lead to trigger the inflammatory process [117]. The disorder of inflammatory pathways play a vital role in cancer development [118]. It is also responsible for the incidence of various adverse effects following chemotherapy. [32] The use of doxorubicin during cancer chemotherapy can induce heart inflammation [119, 120]. Furthermore, oxidative stress induced by doxorubicin can affect LPO and activate lysosomal enzymes which lead to promotion of the inflammation in heart tissue [85]. The doxorubicin-treated cardiac cells showed an increased production of proinflammatory medicators such as ROS, NF-*κ*B, COX-2, TNF-*α*, INF-*γ*, TGF-*β*, IL-1*β*, IL-6, and IL-18 levels [56, 63, 68, 121]. NF-*κ*B, as a proinflammatory transcription factor, has a key role in the activation of TNF-*α*, IL-1*β*, IL-1, IL-2, IFN*γ*, COX-2, and iNOS [122–124]. These inflammatory cytokines are able to induce remarkable pathological changes in the form of transmural myocarditis, biventricular fibrosis, and cardiomyopathy [125]. IL-1 is a cytokine well associated to chronic and acute inflammation and other chronic diseases such as cardiomyopathy. It has been also reported that IL-1*β*, one of the members of the IL-1 family, exacerbates myocardial injures in cancer patients treated with chemotherapy drugs; hence, pharmacological inhibition of IL-1*β* can be considered as a promising approach for reduction of chemotherapy-induced adverse cardiovascular events [126]. TGF-*β* is a profibrogenic cytokine that mediates several aspects of the fibrotic process; for instance, it can induce fibroblast proliferation and transformation to myofibroblasts, causing the deposition of collagen and extracellular matrix protein [127, 128]. Additionally, TGF-*β* can modulate cell proliferation, differentiation, apoptosis, and migration [129].

Several studies have reported that curcumin can be suggested as a promising anti‐inflammatory agent [40, 130]. Curcumin, through its anti-inflammatory activities, can decrease the resistance of cancer cells to chemotherapeutic drugs and also protect the normal cells against chemotherapy-induced side effects [32]. According to the finding obtained from previous studies, it was shown that curcumin cotreatment alleviates the doxorubicin-induced cardiac inflammation. In details, it can be mentioned that combined treatment of curcumin and doxorubicin declines the elevated levels of NF-*κ*B, COX-2, TNF-*α*, INF-*γ*, IL-1*β*, IL-6, and IL-18 in the cardiac cells of the doxorubicin-treated animals [56, 62, 68]. Furthermore, the findings of histological examinations demonstrated that doxorubicin-induced cardiac inflammation is mitigated by curcumin coadministration [2, 57–62, 68].

## 5. Perspective of Future Research

Because of its potent anticancer activities, doxorubicin is widely applied to treat cancer patients; however, irreversible cardiotoxic effects of this chemotherapeutic drug have limited its clinical applications. The published data demonstrated that using the chemoprotective agent of curcumin can alleviate the doxorubicin-induced cardiotoxicity. The researchers have reported several mechanisms for cardioprotective effects of curcumin against doxorubicin-induced cardiotoxicity, including antioxidant, antiapoptosis, anti-inflammatory, and so on. In addition to its chemoprotective, curcumin through chemosensitizer effects can sensitize cancer cells to chemotherapy drugs.

The findings obtained for cardioprotective effects of curcumin against doxorubicin-induced cardiotoxicity are based on nonclinical studies (in vitro and in vivo models). Therefore, suggesting the use of curcumin in cancer patients as a cardioprotector agent against cardiotoxicity mediated by doxorubicin or other chemotherapeutic drugs requires further clinical studies because sometimes the findings may be different between the in vitro and in vivo models and clinical studies.

## 6. Limitations

There are several limitations which should be addressed: (1) remarkable heterogeneity was encountered perhaps due to various regimens, doses, duration, center settings, and populations enrolled, calling for cautious interpretation of the data, (2) several studies suffer from significant sources of bias, and (3) the effects of doxorubicin treatment alone or in combination with curcumin on the cardiac cells/tissue in many occasions were assessed by very few studies; hence, the evidence to support it is low.

## 7. Conclusion

The data presented in this systematic review reveal that the doxorubicin chemotherapeutic agent induces biochemical and histological changes on the cardiac cells/tissue, leading to cardiac adverse effects. It has been also shown that the curcumin cotreatment alleviates the doxorubicin-mediated cardiotoxicity. Mechanically, curcumin exerts its cardioprotective effects through several main mechanisms, such as antioxidant, antiapoptosis, and anti-inflammatory.

## Figures and Tables

**Table 1:** The characteristics of included studies.

Author and year	Model	DOX dosage and protocol of usage; administration route	Outcomes of DOX on cardiac cells/tissue	Curcumin dosage and protocol of usage; administration route	Curcumin coadministration outcomes
Venkatesan, 1998 [65]	In vivo/rats	30 mg/kg and single dose; i.p.	↑creatine kinase and LDH, ↑LPO, ↑TBARS, ↓GSH and GPx, ↑catalase, ↑conjugated dienes	200 mg/kg and seven days prior and two days after DOX injection; NI	↓creatine kinase and LDH, ↓LPO, ↓TBARS, ↑GSH and GPx, ↓catalase, ↓conjugated dienes

Shah et al., 2008 [66]	In vivo/rats	10 mg/kg and single dose; i.v.	↑CPK and LDH, ↑SGOT, ↑LPO, ↓SOD, GSH, and catalase, ↓membrane bound enzymes (Na^+^/K^+^ ATPase, Mg^+2^ ATPase, and Ca^+2^ ATPase)	80 mg/kg/day and for 30 days followed by DOX injection on the 30^th^ day; oral	↓CPK and LDH, ↓SGOT, ↓LPO, ↑SOD, GSH, and catalase, ↑membrane bound enzymes (Na^+^/K^+^ ATPase, Mg^+2^ ATPase, and Ca^+2^ ATPase)

Mohamad et al., 2009 [67]	In vivo/rats	7.5 mg/kg and single dose; i.v.	↑creatine kinase and LDH, ↑troponin-T	200 mg/kg/day and 10 days before DOX injection and then was continued for next 20 days.	↓creatine kinase and LDH, ↓troponin-T

Hosseinzadeh et al., 2011 [55]	In vitro/H9c2 cells	0.5, 1.5, 3, and 6 *μ*M and 22 h	↓cell viability, ↑caspase-3	0.5, 1, 1.5, 3, 5, 10, and 15 *μ*M and 1 h prior to DOX injection	↓↓cell viability (for 5, 10, and 15 *μ*M), ↑↑caspase-3 (for 10 and 15 *μ*M), ↓cIAP1 mRNA expression

Hosseinzadeh et al., 2011 [56]	In vitro/H9c2 cells	3 *μ*M and 6 h	↓cell viability, ↑ROS, ↑apoptosis, ↑BAX, ↓Bcl-2, ↑BAX to Bcl-2 ratio, ↑caspase-9, ↑NF-*κ*B	5, 10, and 15 *μ*M and 1 h prior to DOX injection	↓↓cell viability, ↑↑ROS, ↑↑apoptosis, ↑↑BAX (for 15 *μ*M), ↓↓Bcl-2, ↑↑BAX to Bcl-2 ratio, ↑↑caspases-8 and 9 (for 10 and 15 *μ*M), ↓NF-*κ*B

Sadzuka et al., 2012 [69]	In vivo/mice	15 mg/kg and on the second day; i.p.	↑serum AST and ALT, ↑LPO, ↓GPx	100 mg/kg/day and for 5 days; i.p.	↓serum ALT, ↓LPO, ↑GPx

Swamy et al., 2012 [58]	In vivo/rats	Cumulative dose of 15 mg/kg and 2.5 mg/kg in six equal injections for two weeks; i.p.	↑mortality, ↓bodyweight, ↑CPK and LDH, ↑AST, ALT and ALP, ↑MDA, ↓GSH, SOD, and catalase, induction of histological changes (loss of myofibrils and vacuolization of cytoplasm)	200 mg/kg/day and as a pretreatment and for two weeks; oral	↓mortality, ↑bodyweight, ↓CPK and LDH, ↓AST, ALT, and ALP, ↓MDA, ↑GSH, SOD, and catalase, alleviation of DOX-induced histological changes

Imbaby et al., 2014 [59]	In vivo/rats	Cumulative dose of 18 mg/kg and 1.5 mg/kg in 12 equal injections for 23 days (every other day); i.p.	↓bodyweight (%), heart index, and survival rate, ↑troponin I, CPK, and CK-MB, ↑MDA and NO, ↓GPx and SOD, ↓intact form of mitochondrial DNA, ↓mitochondrial DNA content, ↑fragmentation of nuclear DNA, induction of histological changes (↑degeneration of cardiac muscle fibers along with marked vacuolization of cytoplasm, interstitial edema, ↑injured cells)	100 and 200 mg/kg/day and for 30 days and starting one week before DOX treatment; oral	↑bodyweight (%) and survival rate (for 200 mg/kg), ↓troponin I, CPK, and CK-MB levels (for 200 mg/kg), ↓MDA and NO (for 200 mg/kg), ↑GPx and SOD (for 200 mg/kg), ↑intact form of mitochondrial DNA (for 200 mg/kg), ↑mitochondrial DNA content (for 200 mg/kg), ↓fragmentation of nuclear DNA (for 200 mg/kg), alleviation of DOX-induced histological changes (mild degeneration with minimal vacuolization of the cytoplasm, mild interstitial edema, slight separation, ↓injured cells)

Sheu et al., 2015 [64]	In vitro/3T3 normal fibroblast cells	1.5 *µ*M and 1, 3, 5, and 24 h	↑ROS, ↑apoptosis, ↑mitochondrial superoxide generation	10, 20, and 30 *µ*M and concomitant and 5 h prior to DOX treatment	↓ROS (for 30 *µ*M and 5 h prior to DOX treatment), ↓apoptosis (for 30 *µ*M and 5 h prior to DOX treatment), ↓mitochondrial superoxide generation (for 30 *µ*M and 5 h prior to DOX treatment)

Junkun et al., 2016 [70]	In vitro/H9c2 cells	1 *μ*mol/L and 0, 1, 3, 6, and 24 h	↑MDA, ↓GSH and SOD, ↑apoptosis, ↑loss of mitochondrial transmembrane potential, ↑PiC (Slc25a3) expression	10, 12, and 15 mg/L and concomitant and 2 h prior to DOX treatment	↓MDA, ↑GSH and SOD, ↓apoptosis, ↓loss of mitochondrial transmembrane potential, ↓PiC (Slc25a3) expression

Benzer et al., 2017 [68]	In vivo/rats	40 mg/kg and single dose on the 5^th^ day; i.p.	↑CK-MB, LDH and troponin I, ↓SOD, catalase, GSH and GPx, ↑MDA, ↑8-OHdG and 3,3′-dityrosine expression, ↑NF-*κ*B, TNF-*α* and IL-1*β*, ↑iNOS and COX-2, ↑caspase-3, severe hemorrhage and mononuclear cell infiltrates	100 and 200 mg/kg/day and for 7 days; oral	↓CK-MB, LDH, and troponin I, ↑SOD, catalase, GSH, and GPx, ↓MDA, intermediate 8-OHdG and 3,3′-dityrosine expression, ↓NF-*κ*B, TNF-*α*, and IL-1*β*, ↓iNOS and COX-2, ↓caspase-3, moderate interstitial hemorrhage

He et al., 2018 [57]	In vitro/cardiomyocytes (from rats) and in vivo/mice	1 *μ*M and for 24 h (for in vitro) and cumulative dose of 15 mg/kg and 2.5 mg/kg in 6 equal injections over 3 weeks; i.p. (for in vivo)	↓cell viability, ↑LDH and creatine kinase, ↓catalase, SOD, and GPx, ↑MDA, ↑ROS, ↑MMP loss, ↑mPTP opening, ↑caspase-3, ↑apoptosis, ↑14-3-3*γ* expression, ↑bad phosphorylation, ↑Bcl-2, severe inflammatory changes in the myocardial tissue (inflammatory infiltration, cellular swelling, spotted necrosis, small necrosis, and interstitial cell hypertrophy)	5, 10, 20, and 40 *μ*M and for 22 h prior to Dox treatment (for in vitro) and 50 mg/kg/day and for 6 consecutive weeks (starting 3 weeks before DOX treatment); i.g. (for in vivo)	↑cell viability, ↓LDH and creatine kinase, ↑CAT, SOD and GPx, ↓MDA, ↓ROS, prevention of MMP loss, ↓mPTP opening, ↓caspase-3, ↓apoptosis, ↑↑14-3-3*γ* expression, ↑↑Bad phosphorylation, ↑↑Bcl-2, alleviation of myocardial injury

Jain and Rani, 2018 [9]	In vitro/H9C2 cells	0.1, 0.5, 1, and 1.5 *μ*M and 48 h	↓cell viability	10 *μ*M and concomitant	↑cell viability

Jain and Rani, 2018 [2]	In vitro/H9C2 cells	15 *μ*M and 24 h	Induction of morphological alterations, ↑ROS, ↑SOD and catalase, ↓mitochondrial membrane integrity, ↑caspases-2, 3, and 9, ↑cellular death, ↓Bcl-2, ↑BAX	20 *μ*M and concomitant and 24 h prior to DOX treatment	Prevention of Dox-induced morphological alterations (for pretreatment mode), exaggeration of Dox-induced morphological alterations (for concomitant mode), ↓ROS (for pretreatment mode), ↑↑ROS (for concomitant mode), ↑↑SOD and catalase (for pretreatment mode), ↓SOD and catalase (for concomitant mode), ↑mitochondrial membrane integrity (for pretreatment mode), ↓↓mitochondrial membrane integrity (for concomitant mode), ↓caspases-2, 3, and 9 (for pretreatment mode), ↑↑caspases-3 and 9 activities (for concomitant mode), ↓cellular death (for pretreatment mode), ↑↑cellular death (for concomitant mode), ↑Bcl-2 (for pretreatment mode), ↓↓Bcl-2 (for concomitant mode), ↓BAX (for pretreatment mode), ↑↑BAX (for concomitant mode)

Jafarinezhad et al., 2019 [61]	In vivo/rats	4 mg/kg/injection and on days 1, 8, 15, and 22; i.p.	↓bodyweight, ↓ventricle weight and volume, ↑troponin-I, ↓myocardium volume, ↓number of cardiomyocyte nuclei, ↑connective tissue volume and cardiomyocyte volume, induction of histological changes (atrophic changes in myocardium and vessels, reduction in cardiomyocyte population, fibrous tissue formation, hypertrophy of cardiomyocytes)	100 mg/kg/day and for 24 days; oral	↑bodyweight, ↑ventricle weight and volume, ↓↑troponin-I, ↑myocardium volume, ↑number of cardiomyocyte nuclei, ↓connective tissue volume and cardiomyocyte volume, alleviation of DOX-induced histological changes

Wu et al., 2019 [62]	In vivo/rats	Cumulative dose of 21 mg/kg and 3 mg/kg for seven times in two weeks; i.v.	↓bodyweight, ↑creatine kinase, CK-MB, and LDH, ↑INF-*γ*, TNF-*α*, IL-6, and IL-1*β*, ↑MCP-1 and Egr1, ↑apoptosis, ↑caspase-3 and BAX, ↓Bcl-2, induction of histological changes (intracellular edema and cytoplasmic vacuolization, necrosis, inflammatory cell infiltration, wavy degeneration of cardiac muscle fibers, damaged mitochondria, marked extracellular space expansion and swollen)	100 mg/kg/day and seven days prior to the first DOX injection and for four weeks; oral	↑bodyweight, ↓creatine kinase, CK-MB and LDH, ↓INF-*γ*, TNF-*α*, IL-6, and IL-1*β*, ↓MCP-1 and Egr1, ↓apoptosis, ↓caspase-3 and BAX, ↑Bcl-2, alleviation of DOX-induced histological changes

Yadav et al., 2019 [60]	In vivo/rats	Cumulative dose of 15 mg/kg and 2.5 mg/kg in 6 equal injections for two weeks; i.p.	↑mortality, ↓heart weight, bodyweight, and heart weight to bodyweight ratio, ↑CK-MB, ALT, AST, LDH and ALP, ↑CPK and MDA, ↓SOD, GSH, and catalase, loss of myofibrils, and vacuolization of the cytoplasm	200 mg/kg/day and for two weeks; oral	↓mortality, ↑heart weight, bodyweight, and heart weight to bodyweight ratio, ↓CK-MB, ALT, AST, LDH, and ALP, ↓CPK and MDA, ↑SOD, GSH, and catalase, ↓loss of myofibrils and vacuolization of the cytoplasm

Yu et al., 2020 [63]	In vitro/H9C2 cells and in vivo/mice	2 *µ*mol/L and 24 h (for in vitro) and cumulative dose of 24 mg/kg and 3 mg/kg every other day; i.p. (in vivo)	↓bodyweight, heart weight, and heart to bodyweight ratio, ↑creatine kinase, LDH, and AST, ↑MDA, ↑BAX, ↑BAX to Bcl-2 ratio, ↑NLRP3, caspase-1 and IL-18, ↑Beclin1 and LC3II to LC3I ratio, ↓phosphorylation of Akt and mTOR, ↑troponin-I, ↑O_2_^−^ level, ↑cells with GFP-LC3 autophagosomes (%)	10 *µ*mol/L and concomitant (for in vitro) and 50, 100, 200 and 400 mg/kg/day; i.g. (in vivo)	↑bodyweight, heart weight, and heart to bodyweight ratio, ↓creatine kinase, LDH and AST, ↓MDA (for 200 and 400 mg/kg), ↓BAX, ↑Bcl-2 (for 100 and 200 mg/kg), ↓BAX to Bcl-2 ratio, ↓caspase-1 (for 100 and 200 mg/kg), ↓Beclin1, ↓LC3II to LC3I ratio (for 100 and 200 mg/kg), ↑phosphorylation of Akt, ↑phosphorylation of mTOR (for 100 and 200 mg/kg), ↓troponin-I (for 100 mg/kg), ↓O_2_^−^ level, ↓cells with GFP-LC3 autophagosomes (%)

↑, increase; ↓, decrease; NI, not informed; i.p., intraperitoneal; i.g., intragastrical; DOX, doxorubicin; MDA, malondialdehyde; ROS, reactive oxygen species; GPx, glutathione peroxidase; SOD, superoxide dismutase; MMP, matrix metalloproteinase; BAX, Bcl-2-associated X protein; Bcl-xL, B cell lymphoma-extra large; IL-1*β*, interleukin 6; TNF-*α*, tumor necrosis factor alpha; LPO, lipid peroxide; LDH, lactate dehydrogenase; AST, aspartate aminotransferase; ALT, alanine aminotransferase; ALP, alkaline phosphatase; CPK, creatine phosphokinase; GSH, glutathione; CK-MB, creatine kinase-myocardial band; TBARS, thiobarbituric acid reactive substances; CPK, creatine phosphokinase; TGF-*β*1, transforming growth factor beta 1; mTOR, mammalian target of rapamycin; iNOS, inducible nitric oxide synthase; SGOT, serum glutamate oxaloactetate transaminase; mPTP, mitochondrial permeability transition pore; cIAP1, cellular inhibitor of apoptosis protein-1; INF-*γ*, interferon gamma; MCP-1, monocyte chemoattractant protein-1; Egr1, early growth response protein-1; NLRP3, NLR family pyrin domain containing 3; NF-*κ*B, nuclear factor-kappa B; NO, nitric oxide; COX-2, cyclooxygenase-2.

## Data Availability

The data used to support the findings of this study are available from the corresponding author upon request.
